# The Structure of FeAl Sinters Fabricated Using Cyclic Loading

**DOI:** 10.3390/ma8020575

**Published:** 2015-02-09

**Authors:** Tomasz Durejko, Michał Ziętala, Zbigniew Bojar

**Affiliations:** Department of Advanced Materials and Technologies, Faculty of Advanced Technologies and Chemistry, Military University of Technology, Kaliskiego 2 Str., 00-908 Warsaw, Poland; E-Mails: mzietala@wat.edu.pl (M.Z.); zbojar@wat.edu.pl (Z.B.)

**Keywords:** iron aluminides, sintering process, cyclic loading

## Abstract

A two stage process including a sintering under a cyclic loading is proposed as an alternative fabrication method of dense FeAl intermetallics from elemental powder mixtures. The first stage (pre-sintering) is conducted at two temperature values (620 °C and 670 °C, respectively) under a static and a cyclic loading with a frequency of 20, 40 and 60 Hz. The second one includes a pressureless sintering at temperature of 1250 °C, under a protective argon atmosphere. A suitable selection of pre-sintering parameters (temperature, type and frequency of pressing) allows approximately five times grain size reduction of FeAl phase in comparison to particle size of raw Fe and Al powder material (40–60 µm), as well as induces an effective fragmentation of oxide layers. For the sinters obtained using 60 Hz loading frequency an oxide particle size of 4.0 or 4.5 µm (smaller for sintering with liquid phase) is observed. Material obtained after the full heat treatment are characterized by a fine-grained structure of chemically homogeneous FeAl phase with uniformly distributed Al_2_O_3_ spherical particles along grain boundaries. Moreover, it was found that temperature and frequency of loading during the presintering process also affect a consolidation level of the Fe-Al powder mixture, which increases with rising both temperature and frequency.

## 1. Introduction

Intermetallic phases were recognized as a minor component of classic structural alloys over 60 years ago. However, an excessive study on intermetallics, *i.e.*, alloys with dominating fraction of these phases, has been started in the last decade of the 20th century. A much research effort has been focused on a technology-structure-properties relationship of intermetallic phases that are formed between aluminum and transition metals (e.g., Fe-Al [1,2,3] or Ni-Al [4,5]). A great interest has been devoted to phases from the Fe-Al binary system with a special emphasis on a FeAl intermetallic phase. However, there are a few disadvantages that limit its applications, namely: a low plasticity, a tendency to brittle cracking at ambient temperature, and a low creep resistance in elevated temperatures [6].

Solutions of these problems are researched both in the terms of structure modification and production technology. Actually applied FeAl fabrication methods, e.g., a forging, a rolling and a hot extrusion (at temperature even up to 1260 °C) [1,2], a laser deposition [7,8] or a gas detonation [9,10] are complex, technologically difficult, and expensive. The existed “technological gap” can be filled by techniques based on a powder metallurgy (PM). From the economical point of view, the PM is the most advantageous method that simultaneously allows producing complex components for application in various industries like: aircraft, automotive, electronics *etc*. Moreover, these technologies give a possibility of a precise control of microstructure of sinters and often eliminate necessity of the final treatment of produced components [11]. Unfortunately, the sintering process of FeAl alloyed powder [12] requires a high temperature and a high consolidation pressure [13]. Compaction/sintering methods mainly based on an isostatic hot pressing or a hot extrusion belong to expensive and complex technological solutions. A big chance for changing this situation could be an implementation of FeAl intermetallics production via techniques based on metallurgy of technically pure iron and aluminum powders [14,15,16]. Gedevanishvili and Deevi [17] discussed in details the problem of FeAl sinters obtained by technically pure elemental powders. They stated that during a heating of the iron and aluminium mixture with a 60 to 40 at.%. ratio, two exothermic reactions take place, being crucial for the final structure of the sinter. They also noticed that a heating rate is one of the key factors influencing the kind of phase transitions and a final density during formation of the Fe-Al intermetallic phases from elemental components, what was also confirmed by Karczewski and Jozwiak [18]. However, the authors did not analyze the problem of an oxide formation. It is clear that formation of stable oxide films, mainly Al_2_O_3_ or rather maintenance of existing oxides is a natural side-effect of processing of an iron-aluminum powder mixture into high-aluminum content sinters [19]. Due to an application of cyclically variable loading primary oxide films undergo a fragmentation and do not act as a diffusion barrier, simultaneously playing a role of strengthening phase (depending on a dispersion mode). Furthermore, a control of microstructure (a grain size of FeAl matrix, a size and a distribution of oxide particles) of the final sinter may be provided by a proper selection of process parameters (temperature and pressing pressure frequency) during preliminary sintering stage.

## 2. Results and Discussion

**Figure 1:**
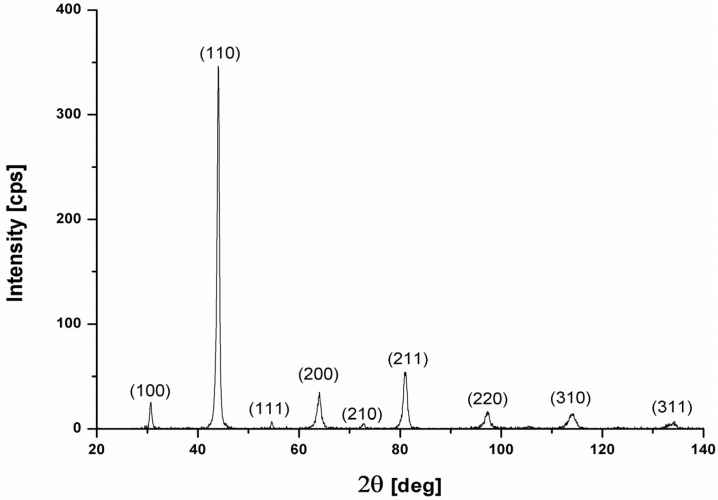
An example of diffraction pattern taken from of the sinter after the full cycle of heat treatment.

Results of the structural evaluation shown that all obtained sinters are characterized by ordered FeAl matrix (Figure 1) with alumina oxides distributed along grains boundary.

**Figure 2:**
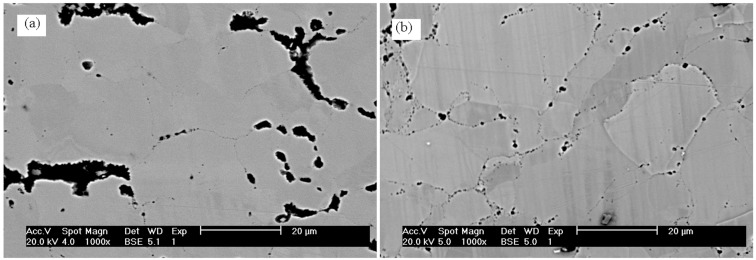
SEM microphotographs of FeAl sinter after the full heat treatment, (**a**) The pre-sintering at 620 °C, 0 Hz; (**b**) the pre-sintering at 670 °C, 60 Hz.

The SEM microphotographs of FeAl sinters after the full heat treatment are shown in Figure 2. It is observed that Al_2_O_3_ particles possess a different morphology related to the applied frequency of loading during the pre-sintering process.

**Figure 3:**
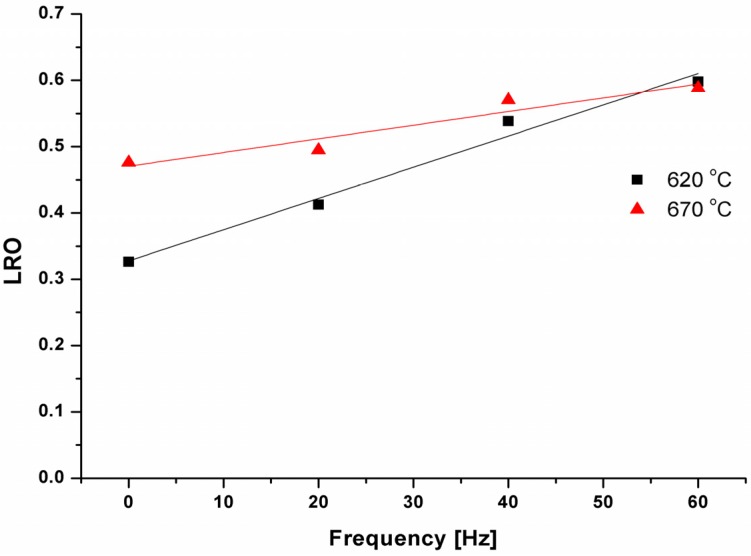
An influence of the pre-sintering parameters on the long-range order (LRO) of FeAl phase.

The analysis of obtained XRD patterns shows that value of the long-range order (LRO) of FeAl matrix depends on applied technological variant. A linear increase of the LRO parameter with an increase of the frequency of loading is observed for samples pre-sintered in the solid phase (at 620 °C) as well as in the liquid phase (at 670 °C). Moreover, the sample produced via the static variant of sintering at 620 °C exhibits the LRO parameter smaller about 0.2 than 0 Hz variant at 670 °C (Figure 3).

Additionally, with increasing of the frequency pressing, a difference between the LRO value calculated for samples pre-sintered at 620 °C and 670 °C variant decreases. Finally, the ordering reached 0.6 for both samples pre-sintered at the highest considered frequency value.

**Figure 4:**
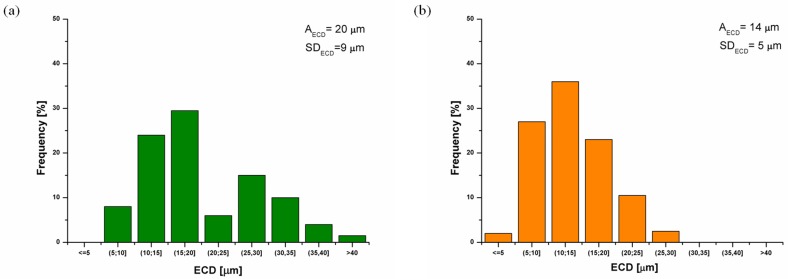
Influence of frequency of cyclic loading on the size and the dimensional homogeneity of the FeAl alloy pre-sintered at 620 °C: (**a**) 20 Hz; (**b**) 40 Hz.

A statistical analysis of the grain size for sinters obtained at 620 °C shows a twice decrease in the equivalent diameter (ECD) as compared to the size of batch powder particles (Table 1). It is also clear that an implementation of the cyclically variable load is advantageous in terms of increasing of the structure refinement. For the loading frequency of 20 Hz a small change in grain ECD value is observed. Simultaneously, a relatively high standard deviation (a spread around the mean ECD value) demonstrates a rise of a grain dimensional inhomogeneity. At 40 Hz load frequency a further grain refinement and a better homogeneity of FeAl matrix are observed (Figure 4). Increasing of the loading frequency to 60 Hz does not lead to a further decrease of the grain size (Table 1).

**Table 1:** Average equivalent diameter of FeAl grains after various technological processes.

Technological variant		FeAl grain size (µm)
Temperature (°C)	Frequency (Hz)	
620 °C	0	24 ± 7
	20	20 ± 9
	40	14 ± 5
	60	14 ± 5
670 °C	0	16 ± 6
	20	13 ± 5
	40	10 ± 5
	60	10 ± 3

However, a further refinement of the matrix structure is possible after increasing temperature of pre-sintering to 670 °C. The alloy produced by the “static” variant is characterized by the mean grain size of 16 μm (as compared to 24 μm for the sample produced via the “static” variant at 620 °C) with the dimensional homogeneity estimated as 6 µm. Starting from 40 Hz loading frequency at temperature 670 °C and 300 MPa pressure, a size of grain in intermetallic phase stabilizes at the level of 10 μm level and for the next technological variant (300 MPa, 60 Hz, 670 °C) becomes more dimensionally homogeneous (Table 1).

**Figure 5:**
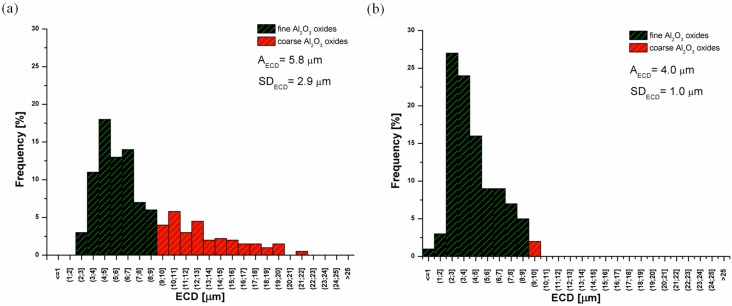
Distribution of Al_2_O_3_ oxides equivalent diameter: (**a**) The pre-sintering at 620 °C, 0 Hz, (**b**) The pre-sintering at 670 °C, 60 Hz.

Results of the analysis of Al_2_O_3_ particles size revealed a presence of two dimensional groups in fabricated sinters (Figure 5). Oxides belonging to the first group, that are dimensionally comparable with ECD values of the FeAl matrix, can be considered as a brittle component (the particles population described as coarse oxides (I-Al_2_O_3_) in Figure 5). On the other hand, the second fraction (II-Al_2_O_3_-fine oxides) that is characterized by a smaller size than matrix grain plays the role of a strengthening phase.

The fraction of alumina particles treated as a harmful phase, was excluded from the statistical analysis for the strengthening phase. The size of II-Al_2_O_3_ oxides shows a slightly decreasing tendency with the increase of the frequency and temperature of the pre-sintering process (Table 2). The best results are obtained for the sample processed with 60 Hz at 670 °C.

**Table 2:** Average equivalent diameter of Al_2_O_3_ oxide particles (strengthening phase).

Technological variant		Al_2_O_3_ oxides size (µm)
Temperature (°C)	Frequency (Hz)	
620 °C	0	5.8 ± 2.9
	20	5.6 ± 1.9
	40	4.8 ± 1.7
	60	4.5 ± 1.6
670 °C	0	5.3 ± 1.6
	20	4.7 ± 1.5
	40	4.2 ± 1.3
	60	4.0 ± 1.0

**Figure 6:**
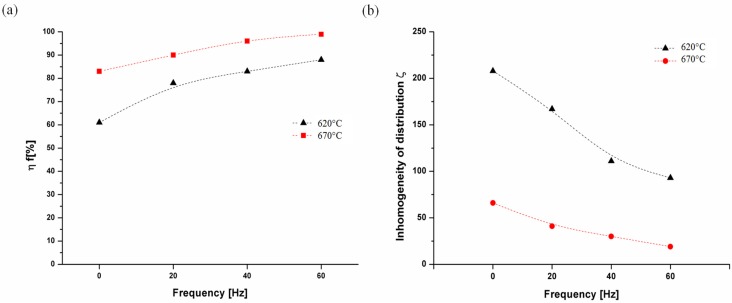
An influence of the loading frequency and the pre-sintering temperature on (**a**) Al_2_O_3_ oxide fragmentation efficiency η_f_, (**b**) The inhomogeneity of oxide phase spacing ξ.

It is also found that the appropriate control of temperature and the frequency of the consolidation pressure during the pre-sintering leads to a significant change of the harmful oxide fraction in sinters structure. The oxide fragmentation efficiency η_f_ was calculated for investigated samples according to Equation (3) and then plotted as a function of the loading frequency (Figure 6a).

It is found that the oxide fragmentation efficiency equals 61% for the “static” sintering at 620 °C. The increase of the load frequency results with a rise of the η_f_ parameter value (reaching 91% for the sample processed with 60 Hz). An increasing of pre-sintering stage temperature up to 670 °C significantly affects the η_f_ parameter value. The static load gives over 20% higher fragmentation efficiency as compared to the static pre-sintering in solid state. A change in the loading type leads to an intensive oxides fragmentation—for samples fabricated with 40 Hz and 60 Hz the η_f_ parameter reaches almost 100% which means that coarse oxides particles are eliminated.

The analysis of the efficiency of oxides fragmentation was also supplemented by an evaluation of a homogeneity of the oxide phase distribution in the FeAl intermetallic matrix, which is a very important parameter for such type of a composite structure. Performed calculations point toward an advantageous influence of the cyclic loading on the homogeneity of the Al_2_O_3_ particles distribution (Figure 6b). A value of the inhomogeneity parameter calculated for the material pre-sintered at 620 °C decreases significantly with raising the load frequency. However, for all considered frequency values it is apparently larger than the inhomogeneity of oxide particles distribution obtained for the material pre-sintered at 670 °C. It is worth noting that inhomogeneity of the Al_2_O_3_ particles distribution for samples preliminary sintered with the presence of the liquid phase is only slightly dependent on the applied load frequency.

Results of the porosity examinations revealed that the cyclically variable load has also a hard impact on a density of fabricated sinters. The porosity calculated by planimetric method for the material pre-sintered at temperature of 620 °C and pressed with the static loading is 3.4% (Table 3). The application of 20 Hz load frequency results with a decrease of porosity to about 2.3%. A further increase in the frequency or temperature has an insignificant effect on the analysed parameter. The sinters obtained at 620 °C exhibit a stronger correlation between a density and a type of loading than those manufactured with a presence of the liquid phase. Moreover, it is observed that 60 Hz and 40 Hz variants give the same porosity value for both temperature values of the pre-sintering. It is also found that the porosity calculated by the Archimedes method gives approximately twice higher results than those calculated by the planimetric method.

**Table 3:** The FeAl sinters porosity calculated by planimetric and Archimedes methods.

Porosity (%)	Pre-sintering temperature (°C)							
	620				670			
	Frequency (Hz)							
	0	20	40	60	0	20	40	60
Stereological method	3.4	2.3	2.1	2.1	2.2	1.9	2.1	2.1
Archimedes’ method ^1^	6.1	5.4	5.3	4.8	5.0	4.9	4.8	4.7
Archimedes’ method ^2^	4.3	3.6	3.5	3.1	3.2	3.1	3.0	2.9

^1^: theoretical density of Fe40Al alloy as a reference value–6.06 g/cm^3^; ^2^: a high quality cast Fe40Al alloy as a reference material–5.95 g/cm^3^, carried out at the same measuring conditions as for sintered samples.

However, results obtained by these two methods are more consistent when a high quality cast of Fe40Al alloy is used as a reference sample in the planimetric method. Finally, it is stated that FeAl sinters porosity does not exceed 3% (except for the sample fabricated by the static variant at 620 °C).

## 3. Experimental Section

Firstly, technically pure iron (99.7%) and aluminium (99.5%) manufactured by ABCR GmbH & Co KG company (Karlsruhe, Germany) were separated using Analysette Pro 3 vibratory sieve shaker in order to obtain 40 ÷ 60 µm fractions for each powder. A mixture of technically pure Fe and Al powders (with a stoichiometric composition of 60% iron and 40 at.%. aluminum) was homogenized in Uni Ball 5 ball mill at 30 rpm for twenty minutes.

**Figure 7:**
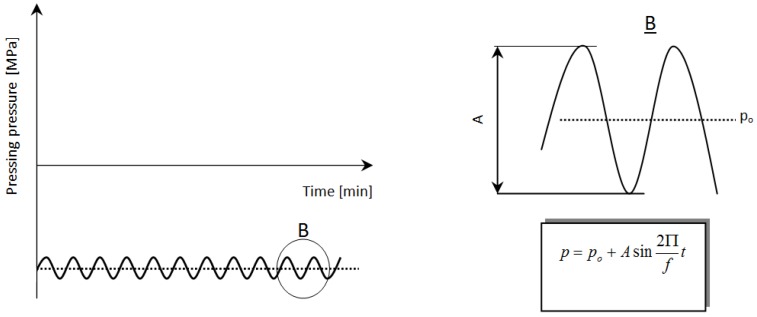
A schematic presentation of cyclic changes of the pressing pressure; where: *p_o_*: assumed level of pressing pressure (MPa); *A*: amplitude of the pressing pressure (MPa); *f*: frequency of the pressing pressure changes (Hz); *t*: time (min).

The first stage (the pre-sintering) of sintering process was based on an uniaxial compression of the prepared powder mixture under a static or a cyclically variable loading at elevated temperature (620 °C and 670 °C) carried out on Instron hydraulic pulsator. A sinusoidal variation of the consolidation pressure (Figure 7) with fixed amplitude of 20 MPa, a mean pressure of 300 MPa and frequency 20, 40 and 60 Hz were selected upon the compression step.

The second stage (pressureless sintering) allowed transforming a multiphase structure of the preliminary compact (with, e.g., a presence of pure iron and Fe_2_Al_5_ phase [20]) into a monolithic structure of the FeAl based solution solid.

Samples for structural tests were cut off by electrodischarge machining and then subjected to a sample preparation routine. The metallographic prepartion included a mechanical grinding on the STRUERS PLANOPOL 3 device (Ballerup, Denmark) using abrasive papers with 100 ÷ 2000 granulation followed by a mechanical polishing on the diamond slurry of the 6, 3, 1 and 0.25 μm granulation. A microstructure of the tested sinters after the full heat treatment was revealed via a chemical etching using the 33% CH_3_COOH + 33% HNO_3_ + 33% H_2_0 + 1% HF reagent. A stereological analysis was carried out on at least 1000 objects for each technological variant. Tests were carried out using SIS software (a part of the microscope equipment). The following structural parameters (individual for each object) were analyzed: an equivalent circle diameter ECD

(1) $$\text{ECD}=\sqrt{\frac{4\text{A}}{\text{Π}}}$$

An oxides spacing inhomogeneity ξ coefficient (calculated for oxides only):

(2) $$\text{ξ}=\left|\frac{{\text{σ}}_{\text{j}}^{2}-{\text{σ}}^{2}}{{\text{σ}}_{\text{j}}^{2}}\right|$$

where: ${\text{σ}}_{j}^{2}$ is the variance of the mean distance between particles for the homogeneous microstructure (${\text{σ}}_{j}^{2}$ = 0.10 [19] was adjusted); σ^2^ is the variance of the mean distance between particles for the real microstructure.

In order to find the oxides fragmentation efficiency (as a result of cyclic loading application) the fragmentation efficiency η_f_ coefficient was calculated as follows:

(3) $$\eta_{\text{f}}=\left(1-\frac{{\text{A}}_{\text{I}}}{{\text{A}}_{\text{II}}}\right)\times 100\%$$

where: A_I_ is the area occupied by coarse oxide particles of the comparable (and larger) size than equivalent diameter of the FeAl grains (the phase is regarded as non-metallic inclusion); A_II_ is the area occupied by fine oxide particles of a smaller size than equivalent diameter of the FeAl grains (particles are regarded as strengthening phase in further works).

A phase composition analysis of samples after the main sintering process was conducted on Seifert XRD 3003 X-ray diffractometer (Ahrenburg, Germany) equipped with a copper target. The analysis was conducted within 20°–140° diffraction angle range with a 0.02° step size and count time of 2 s per step.

Measurements of sinters density were done by Archimedes method using WPS200 laboratory balance. Acetone (CH_3_COCH_3_) was applied as a test liquid. Before measurements each sample was cleaned in an ultrasonic bath for 5 min in acetone. After the chemical cleaning the samples were dried in flow with compressed air. Obtained results were compared with the porosity of sinters calculated by the stereological method.

## 4. Conclusions

The analysis of both a literature data and author’s investigations, carried out for more than 7 years, shows that due to an extremely low plasticity and a tendency to intergranular cracking at ambient temperature of the FeAl cast alloys—the powder metallurgy processing of technically pure iron and aluminum powders is a good alternative for the fabrication of FeAl intermetallics. So far, only a small attention has been paid to the problem of sinters produced via a consolidation of a mixture of non-alloyed powders, and only a few papers has been devoted to the determination of the relationship between a sintering process parameters and FeAl sinters structure.

First of all, the application of the cyclically variable load during the pre-sintering process enables the grain refinement of intermetallic matrix even to 10 µm. Moreover, the continuous alumina layer can be effectively fragmented and homogenously distributed along FeAl grain boundaries. A high efficiency of the Al_2_O_3_ fragmentation process almost completely eliminates coarse oxides. Obtained sinters structure is similar to composite materials, and can be a key to improvement the ductility and the creep resistance of FeAl materials.
